# Increased Beta2-Adrenoceptors in Doxorubicin-Induced Cardiomyopathy in Rat

**DOI:** 10.1371/journal.pone.0064711

**Published:** 2013-05-31

**Authors:** Nolwenn Merlet, Nicolas Piriou, Bertrand Rozec, Amandine Grabherr, Benjamin Lauzier, Jean-Noël Trochu, Chantal Gauthier

**Affiliations:** 1 l’institut du thorax, Unité Inserm UMR 1087/CNRS UMR 6291, Nantes, France; 2 Université de Nantes, Nantes, France; 3 CHU Nantes, l’institut du thorax, Nantes, France; 4 CHU Nantes, Department of Anaesthesiology, Nantes, France; I2MC INSERM UMR U1048, France

## Abstract

**Background:**

The toxicity of doxorubicin, leading to an irreversible heart failure, limits its use as chemotherapeutic agent. The beneficial effects of early administration of β-blocker were reported in patients with heart failure due to doxorubicin, suggesting an important role of β-adrenoceptors (β-ARs). This study aimed to identify a putative target (β-AR and/or its effectors) at the early phase of a chronic doxorubicin-induced cardiomyopathy (Dox-CM) in a rat model.

**Methodology:**

Dox-CM was induced by six doxorubicin injections (cumulative dose: 15 mg.kg^−1^) and validated by echocardiography and left ventricle (LV) catheterization. The β-AR protein expressions in LV were evaluated by western-blot at days 35 (d35) and 70 (d70) after the first doxorubicin injection. *Ex vivo* cardiac contractility (dP/dt_max_, dP/dt_min_) was evaluated on isolated heart in response to specific β-AR stimulations at d35.

**Results:**

At d35, Dox-CM hearts were characterized by mild LV systolic and diastolic dysfunctions, which were exacerbated at d70. In Dox-CM hearts, β_3_-AR expression was only decreased at d70 (-37±8%). At d35, β_1_-AR expression was decreased by 68±6%, but *ex vivo* β_1_-AR function was preserved due to, at least in part, an increased adenylyl cyclase response assessed by forskolin. β_2_-AR expression was increased both at d35 (+58±22%) and d70 (+174±35%), with an increase of *ex vivo* β_2_-AR response at d35. Inhibition of Gi protein with pertussis toxin did not affect β_2_-AR response in Dox-CM hearts, suggesting a decoupling of β_2_-AR to Gi protein.

**Conclusion:**

This study highlights the β_1_/β_2_-AR imbalance in early Dox-CM and reveals the important role that β_2_-AR/Gi coupling could play in this pathology. Our results suggest that β_2_-AR could be an interesting target at early stage of Dox-CM.

## Introduction

Anthracyclines, like doxorubicin (Dox), epirubicin and daunorubicin, are among the most effective drugs used in chemotherapy for cancer patients. Since the late 60s, Dox is frequently used against a variety of cancers including Hodgkin’s lymphoma [1], soft-tissue sarcomas [2], leukemia and solid tumors. However, Dox administration is limited due to severe cardiotoxic effects leading to dilated cardiomyopathy [3]. Prognosis of heart failure (HF) due to Dox-cardiotoxicity is poor and even worse than ischemic or idiopathic dilated cardiomyopathy. Although several mechanisms have been proposed to describe the mechanisms by which Dox induces cardiotoxicity (generation of free radicals, mitochondrial disruption, alteration of cellular energetic, and initiation of apoptotic cascades), these mechanisms are still not fully understood [4]–[6] and there is no specific treatment for Dox-induced cardiomyopathy (Dox-CM) [6], [7]; treatments classically used for other HFs with systolic dysfunction induce only limited beneficial effects in Dox-CM.

As in different HF etiologies, Dox-CM is characterized by an alteration of adrenergic system [8]. However, at the present time, only few studies have examined the role of cardiac β_1_- and β_2_-adrenoceptor (β-AR) subtypes in the pathogenesis of Dox-cardiotoxicity [9], [10] and only one study, at late-onset Dox-CM, assessed β_3_-AR subtype [11], which is recently described as a new target for some β-blockers such as nebivolol [12], [13]. Despite this lack in experimental data, some clinical studies investigated β-blocker therapies in Dox-CM. Kalay *et al.*, demonstrated that left ventricular (LV) diameters remained constant and diastolic function was better preserved after Dox-treatment in patients receiving carvedilol, compared to placebo [14]. However, Georgakopoulos *et al.*, demonstrated that metoprolol, a β-blocker without antioxidative properties, failed to give cardioprotection in lymphoma-treated doxorubicin patients [15]. It was reported that an early start of treatment with angiotensin-converting enzyme inhibitors (ACEIs), in association or not with β-blockers, both improves myocardial contractility [16] and patients’ prognosis [17]. Although the exact mechanism is still poorly understood, the authors highlighted the importance of an early diagnosis, because a delayed treatment (>6 months after the end of chemotherapy) is inefficient [17].

The aim of the present study was to identify a putative target (β-AR and/or its effectors) involved at the early phase of a chronic Dox-CM in a rat model. This study demonstrated for the first time that β_2_-AR expression was increased from 35 days after the first Dox-injection, this effect was maintained until 70 days after the first Dox-injection, resulting in an increase of β_2_-AR-induced contractility. In addition, β_1_-AR function was preserved, in spite of decreased β_1_-AR protein expression. This discrepancy could be explained by an increase of adenylyl cyclase (AC) expression and/or activity as illustrated by an increased forskolin-induced response in Dox-CM rats.

## Methods

### Ethics Statement

All experiments were performed in accordance with the 1996 Guide for the Care and Use of Laboratory Animals published by the U.S. National Institute of Health. The protocol was approved by the Direction Départementale de la Protection des Populations (agreement number C-44 015) and all efforts were made to minimize suffering.

### Animal Model

One hundred and fifty eight male Sprague-Dawley rats (225–250 g) were purchased from Janvier (Le Genest St Isle, France) and were housed under standard conditions of room temperature, humidity (40–60%) and 12 h light/dark cycle. Food and water were available *ad libitum*.

Doxorubicin (Adriblastine® 50 mg/25 mL, Pfizer, France) was administered intraperitoneally in six equal injections (each containing 2.5 mg.kg^−1^) over a period of two weeks, with a total cumulative dose of 15 mg.kg^−1^ body weight (Dox-CM: n = 100). Age-matched rats injected with saline were used as controls (Ctrl: n = 58). Rats were then bred in animal housing until three weeks after the last injection (day 35 (d35); Ctrl: n = 46, Dox-CM: n = 76) or until eight weeks after the last injection (day 70 (d70); Ctrl: n = 12, Dox-CM: n = 24). At d35, twenty Dox-CM rats and twenty Ctrl rats were randomly selected to be hemodynamically explored by echocardiography-Doppler. Then either rats were hemodynamically assessed by LV catheterization, or rat hearts were removed either to test *ex vivo* cardiac contractile function or to perform biochemical studies. At d70, rats were used to perform echocardiography-Doppler and biochemical studies.

### Echocardiography-Doppler

Transthoracic echocardiography was performed using a commercially available ultrasound system (VIVID7, GE Healthcare, Horton, Norway) equipped with a 10 MHz sectorial probe. Rats were anaesthetized with a gas-mixture of 1% isoflurane (Forene®, Abbott France, Rungis, France) in O_2_. The chest was shaved and the animal was positioned on a heating pad in a supine position. All recordings were monitored under a continuous single-channel electrocardiogram obtained on the imaging system by fixing the electrodes to the limbs. Using two-dimensional imaging, a short axis view of the LV at the level of the papillary muscles was obtained and the two-dimensionally guided M-mode recording through the anterior and posterior walls of the LV was taken as recommended by the American Society of Echocardiography [18]. Then, trans-mitral inflow in pulsed-wave Doppler from apical four chamber view and tissue Doppler imaging (TDI) on basal segments of septal and lateral walls in apical four chamber view were taken as previously described [19]. A cine-loop of LV parasternal short axis view with high frame rate was obtained. All acquisitions were performed by the same operator.

All images were digitally stored on hard disks for off-line analysis (EchoPac Q-analysis software, GE Healthcare). Measurements were made on five cardiac cycles and averaged for each data value. The following parameters were determined as recommended by the American Society of Echocardiography [18]: LV end diastolic and systolic diameters (LVEDD and LVESD), diastolic posterior wall thicknesses (dPWth). LV end diastolic and systolic volumes (LVEDV and LVESV) were calculated from the Teichholz method in order to assess LV ejection fraction (LVEF), whereas LV shortening fraction (LVSF) was calculated from LVEDD and LVESD previously measured. LV diastolic function parameters were derived from pulsed-wave trans-mitral inflow pattern and TDI off-line analyses as previously described [20]: the peak of E wave velocities, the isovolumic relaxation time (IVRT), the mean of peak velocities of basal septal and lateral walls (pulsed wave TDI) during systole (Sa) and in early diastole (Ea) to calculate E/Ea ratio. Radial 2D strain analyses were performed using the 2D speckle-tracking method on every medial myocardial segment [21].

### Left Ventricle Catheterization

LV catheterization was performed, at d35, by a 2F microtip pressure catheter (SPR 838, Millar instruments Inc, Houston, Texas). Anaesthesia maintenance on spontaneously breathing rats was performed with an inhalational anaesthesia system for small animal (TEM anaesthesia, Lormont, France). Isoflurane was delivered through a nose mask at a concentration of 2% volume and 1 L.min^−1^ O_2_ flow to limit hemodynamic repercussion. Body temperature was monitored by rectal probe and maintained constant (37°C) by warming-blanket. The right carotid artery was isolated, ligated at the proximal part and the pressure catheter was inserted in. Signals were recorded using an Analogic/Digital converter (EMKA Technologies, Paris, France), stored and displayed on a computer by the IOX1.5.7 Software System (EMKA Technologies). Data were analysed using Datanalyst software (EMKA Technologies). The following parameters were obtained: LV end diastolic pressure (LVEDP), LV contraction and relaxation velocities (dP/dt_max_ and dP/dt_min_, respectively), the index of LV relaxation constant (Tau).

The animals were thereafter sacrificed by injection of a pentobarbital overdose.

### Western-blot

The expressions of β_1_-AR, β_2_-AR, β_3_-AR, Gsα, Giα_2_ and GAPDH were examined by western-blot, at d35 and d70. Hearts were rapidly isolated and placed in a cold Tyrode solution composed as followed (in mM): NaCl, 137; KCl, 5.4; MgCl_2_, 1.2; Na_2_HPO_4_, 1.2; Hepes, 20; CaCl_2_, 1.0; pH 7.4 (Sigma-Aldrich, St Quentin Fallavier, France). LV and septum free walls were carefully separated and freezed in liquid nitrogen and stored at −80°C until used. LV and septum free walls samples were homogenized in 3 mL of Ripa buffer for 1 g of tissue plus 1X PMSF (Interchim, Montluçon, France) and 2X protease inhibitor cocktail (Roche, Mannheim, Germany). Protein samples (25 µg) were submitted to electrophoresis on a 10% polyacrylamide/sodium dodecyl sulfate gel and then were run 150 min at 20 mA per membrane in TG-SDS buffer (Interchim). Gels and nitrocellulose membranes (Hybond C super membrane, *Amersham*, Saclay, France) were equilibrated in TG-SDS buffer with 20% ethanol, and protein fractions were transferred using an electroblotting apparatus (Bio-Rad, Marnes La Coquette, France). Nonspecific binding was blocked by incubating membranes in 5% non-fat dry milk in Tris-buffered saline (TBS) (200 mM Trizma base, 1.4 M NaCl, pH = 7.5) with 0.1% Tween 20 added (TBS-T) and then membranes were incubated with the primary antibody. Membranes were washed in 5% non-fat dry milk in TBS-T and hybridized with the secondary antibody in 1% non-fat dry milk in TBS-T. Finally, membranes were washed with 5% non-fat dry milk in TBS-T then TBS, and antibody complexes were revealed by the enhanced chemiluminescence detection process (Bio-Rad). Chemiluminescence was visualized using an Amersham ImageQuant RT-ECL camera (GE Healthcare) and band signals were assessed by densitometry with ImageQuant TL software (GE Healthcare). For each lane, a ratio to the corresponding GAPDH band intensity was calculated; the use of GAPDH as reference was validated by checking the abundance stability of GAPDH protein between Ctrl and Dox-CM groups.

Antibody references and conditions used in this study are summarized in Table 1.

**Table 1:** Antibodies used for western-blot.

	Primary antibody		Secondary antibody	
Protein	Description	Dilution	Description	Dilution
**β_1_-AR**	Rabbit polyclonal antibody, Sigma-Aldrich(A-272)	1/2,000	Goat anti-rabbit immunoglobulin G, Santa-Cruz Biotechnology (sc-2054)	1/10,000
**β_2_-AR**	Rabbit polyclonal antibody, AbCam Ltd(ab36956)	1/2,000	Goat anti-rabbit immunoglobulin G, Santa-Cruz Biotechnology (sc-2054)	1/15,000
**β_3_-AR**	Rabbit polyclonal antibody, Santa CruzBiotechnology (sc-50436)	1/5,000	Goat anti-rabbit immunoglobulin G, Santa-Cruz Biotechnology (sc-2054)	1/15,000
**Gsα**	Rabbit polyclonal antibody, Merk Chemicals Ltd(371732)	1/1,000	Goat anti-rabbit immunoglobulin G, Santa-Cruz Biotechnology (sc-2054)	1/10,000
**Giα_2_**	Rabbit polyclonal antibody, Merk Chemicals Ltd(371727)	1/5,000	Goat anti-rabbit immunoglobulin G, Santa-Cruz Biotechnology (sc-2054)	1/10,000
**GAPDH**	Mouse monoclonal antibody, Santa-CruzBiotechnology (sc-32233)	1/10,000	Goat anti-mouse immunoglobulin G, Santa-Cruz Biotechnology (sc-2055)	1/50,000

Primary antibodies were diluted in *TBS-T*, excepted for Giα_2_ protein detection which was diluted in 5% non-fat dry milk in *TBS-T*. All secondary antibodies were diluted in 1% non-fat dry milk in *TBS-T.*

### Whole Heart Contractility

At d35, after removal, hearts were quickly flushed into a cold Tyrode buffer, and then were mounted on a cannula *via* the aorta on a Langendorff apparatus (EMKA Technologies) and perfused with a Krebs-modified solution (in mM: NaCl, 116; KCl, 5; MgSO_4_, 1.1; NaH_2_PO_4_, 0.35; NaHCO_3_, 27; glucose, 10; mannitol, 16; Na-Pyruvate, 2; CaCl_2_, 1.8) continuously oxygenated with a 95% O_2_, 5% CO_2_ gas mixture to maintain a pH 7.4. Hearts were perfused at a constant flow-rate of 14 mL.min^−1^ at 37.0±0.4°C. After heart beatings became regular, left auricle was gently cut off to insert a small latex balloon, connected to a pressure transducer to record LV pressure, into the LV *via* mitral valves. Before starting record, the minimum pressure into the balloon was adjusted to 8–15 mmHg. Cardiac parameters were recorded using IOX1.5.7 software (EMKA Technologies): inotropism, lusitropism and chronotropism were respectively evaluated by measuring the contraction velocity (dP/dt_max_), the relaxation velocity (dP/dt_min_) and the heart rate (HR). After the stabilization period, specific β_1_-, β_2_- or β_3_-AR functions and AC stimulation were assessed by constructing concentration-response curves to different pharmacological agents. Thus, β_1_- and β_2_-AR functions were assessed by perfusing isoproterenol (a non selective β-AR agonist, 10^−9^ to 10^−5^ M) in the presence of 10^−6^ M L-748,337 (a selective β_3_-AR antagonist) associated to 10^−6^ M ICI-118,551 (a selective β_2_-AR antagonist) or to 10^−6^ M CGP-20712A (a selective β_1_-AR antagonist), respectively. β_3_-AR function was assessed by perfusing growing concentrations (10^−9^ to 10^−5^ M) of SR 58611A (a β_3_-AR agonist) and AC response was assessed by perfusing growing concentrations (3.10^−8^ to 10^−5^ M) of forskolin (an AC activator). In order to assess the role of Gi in the β_2_-AR pathway, we used pertussis toxin (PTX) (a selective Gi inhibitor). Classically, PTX is administered to rats two or three days before experiments [22]. However, due to high mortality in Dox-CM rats pre-treated with PTX, we were not able to apply this protocol and used another one consisting in pre-treating heart with PTX (4 µg.L^−1^) in a closed system during 30 min, as described by other groups [23]. Data were analysed using Datanalyst software (EMKA Technologies). After Langendorff experiments, hearts were weighed, as well as LV free walls which were carefully separated from the heart.

### Data Analysis and Statistics

Data are presented as means ± standard error to the mean (sem) of n experiments obtained from n different animals. *In vivo* and *ex vivo* cardiac parameters in baseline as well as protein expressions were compared using Student’s *t* test for unpaired data. Concentration-response curves were compared with a two-way ANOVA (concentration, treatments) for repeated measures followed, when appropriated, by a Bonferroni's multiple comparisons test. Differences were considered significant if *P*<0.05.

### Drugs and Chemicals

ICI-118,551, ((±)-*erythro*-(S*,S*)-1-[2,3-(Dihydro-7-methyl-1*H-*inden-4-yl)oxy]-3-[(1-methylethyl)amino]-2-butanolhydrochloride), CGP-20712A, 1-[2-((3-Carbamoyl-4-hydroxy)phenoxy)ethylamino]-3-[4-(1-methyl-4-trifluoromethyl-2-imidazolyl)phenoxy]-2-propanol dihydro chloride, L-748,337, *N*-[[3-[(2*S*)-2-Hydroxy-3-[[2-[4-[(phenylsulfonyl)amino]phenyl]ethyl]amino] propoxy]phenyl]methyl]-acetamide, forskolin and PTX were obtained from Tocris Bioscience (Bristol, UK), isoproterenol was obtained from Sigma-Aldrich (St Quentin Fallavier, France) and SR 58611A, [(RS)-N-[(25)-7-ethxycarbonylmethoxy-1,2,3,4-tetrahydronapht-2-yl]-(2)-2-(3-chlorophenyl)-2 hydroethanamide hydrochloride] was a generous gift from Sanofi-Synthélabo (Montpellier, France). All drugs were dissolved in distilled water, excepted isoproterenol which was solubilised in 1% acid ascorbic and L-748,337 and forskolin which were solubilised in dimethylsulfoxide (Sigma-Aldrich). The final concentration of the solvent in the organ bath was less than 0.1% v.v^−1^ and was used as controls for the effect of the active drug.

## Results

### Mortality and General Characteristics of Animals

**Figure 1:**
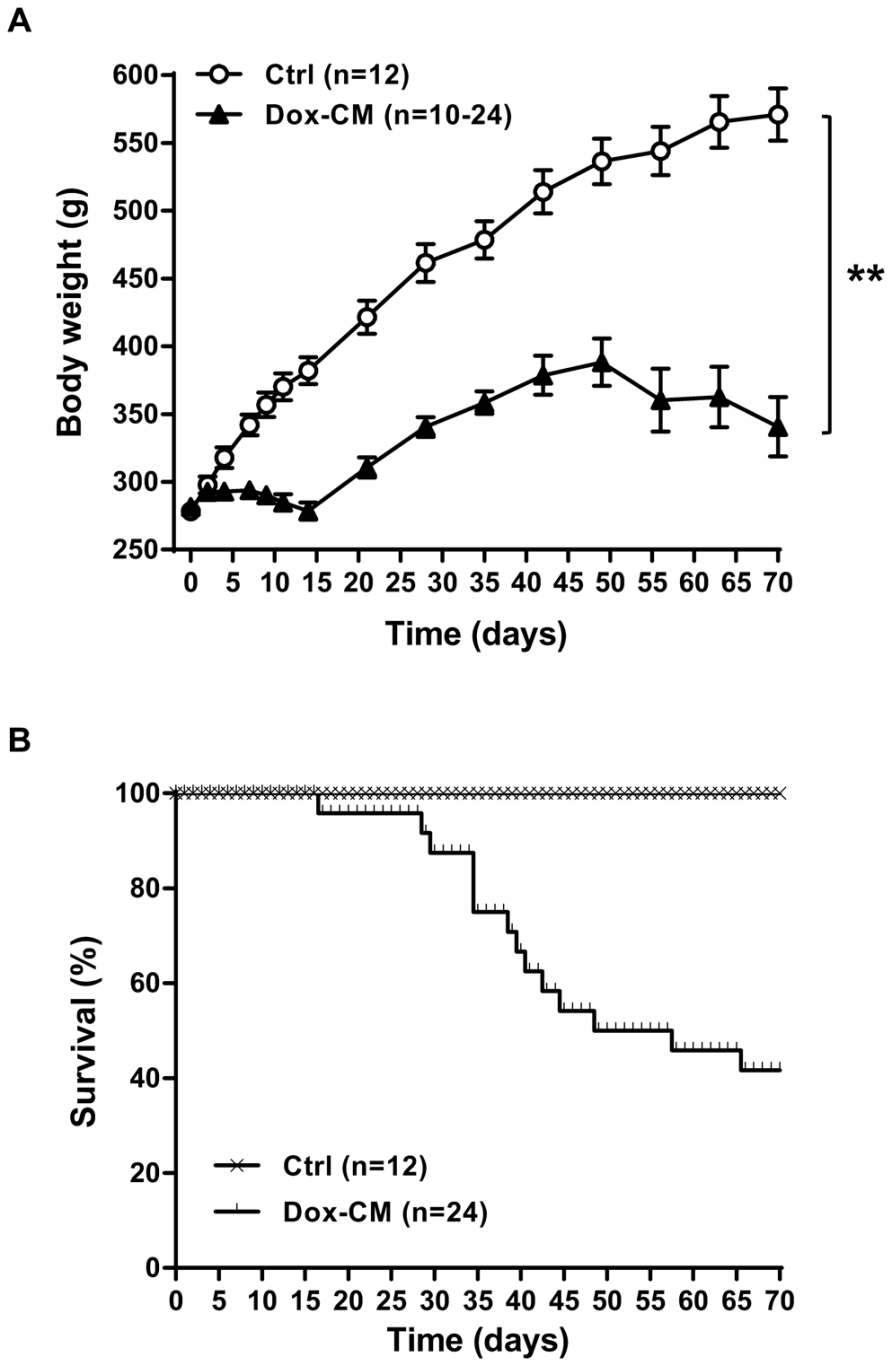
Body weight (A) and survival curves (B) of Ctrl and Dox-CM rats. Values are means ± sem **: *P*<0.001 *vs* Ctrl. Ctrl: control, Dox-CM: Doxorubicin-induced cardiomyopathy.

Dox-CM rats did not gain weight during the Dox-treatment, whereas the body weight of Ctrl rats increased regularly. After the end of Dox-treatment, the rat body weight increased again, but remained significantly lower than those of Ctrl rats, and reached a plateau from 5 weeks after the end of Dox-treatment (day 49; Figure 1A). In addition, Dox-CM rats became lethargic compared to Ctrl one.

The mortality rate was 25.0% at d35 and 58.3% at d70 in Dox-CM when no mortality was observed in Ctrl (Figure 1B). At d35, all surviving Dox-CM rats presented a hepatomegaly and 79.4% of them presented ascites (30.8±5.8 mL), whereas at d70 no hepatomegaly was observed and ascites was present in 38.9% of Dox-CM rats (35.7±4.9 mL). Whole heart and LV weights from Dox-CM rats were significantly smaller than those of Ctrl animals both at d35 and d70, and they were still smaller after normalization to the tibia length. However, heart/body weight ratios and LV/body weight ratios were similar between Ctrl and Dox-CM rats at d35 when it was increased in Dox-CM group compared to Ctrl at d70 (Table 2).

**Table 2:** Cardiac morphological parameters in Ctrl and Dox-CM rats, at d35 and d70.

	d35		d70	
Parameters	Ctrl(n = 30)	Dox-CM (n = 34)	Ctrl (n = 12)	Dox-CM (n = 10)
**Body wt** (g)	516±7	395±6**	571±19	341±22**
**Heart wt** (mg)	1445±31	1112±17**	1317±24	879±51**
**LV wt** (mg)	729±17	562±11**	755±16	483±28**
**Heart wt/Body wt** (mg.g^−1^)	2.80±0.03	2.82±0.04	2.32±0.04	2.60±0.07*
**LV wt/Body wt** (mg.g^−1^)	1.41±0.02	1.43±0.02	1.33±0.04	1.43±0.05
**Heart wt/Tibia length** (mg.cm^−1^)	272±5	218±3**	239±4	166±9**
**LV wt/Tibia length** (mg.cm^−1^)	137±3	110±2**	137±3	92±5**

Ctrl: control; Dox-CM: Doxorubicin-induced cardiomyopathy; LV: left ventricle; wt: weight. Values are means ± sem. *: *P*<0.05 *vs* respective Ctrl. **: *P*<0.001 *vs* respective Ctrl.

### Cardiac Function Obtained by Echocardiography-Doppler

**Figure 2:**
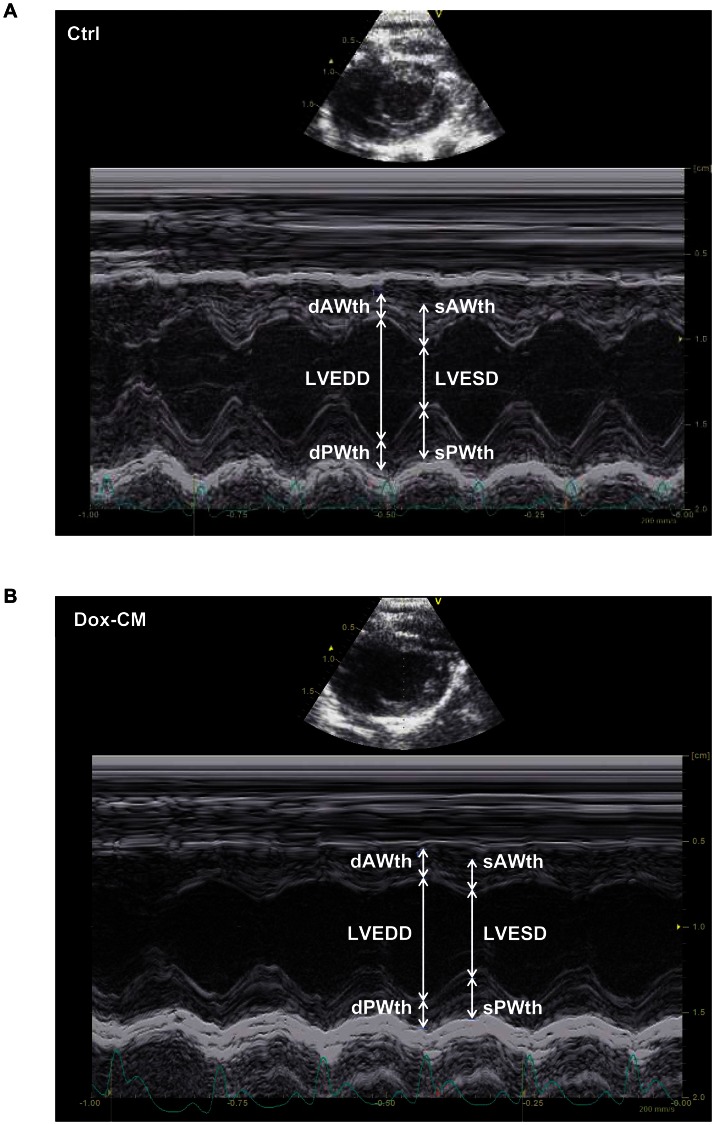
Representative images obtained by echocardiography at day 35 in Ctrl (A) and Dox-CM (B) rats. Images were obtained with a short axis view of a two-dimensionally directed M-mode. Ctrl: control, dAWth: diastolic anterior wall thickness, Dox-CM: Doxorubicin-induced cardiomyopathy, dPWth: diastolic posterior wall thickness, LVEDD: left ventricular end diastolic diameter, LVESD: left ventricular end systolic diameter, sAWth: systolic anterior wall thickness, sPWth: systolic posterior wall thickness.

Echocardiography-Doppler analyses were performed at d35 and d70. Heart rate, measured before all echocardiographic acquisitions, was monitored in order to be similar between both groups (d35: Ctrl: 364±6 bpm, Dox-CM: 371±6 bpm, *P* = 0.427; d70: Ctrl: 368±6 bpm, Dox-CM: 337±17 bpm, *P* = 0.079). Representative images obtained by echocardiography in short axis view of a two-dimensionally directed M-mode at d35 are shown in Figure 2.

**Figure 3:**
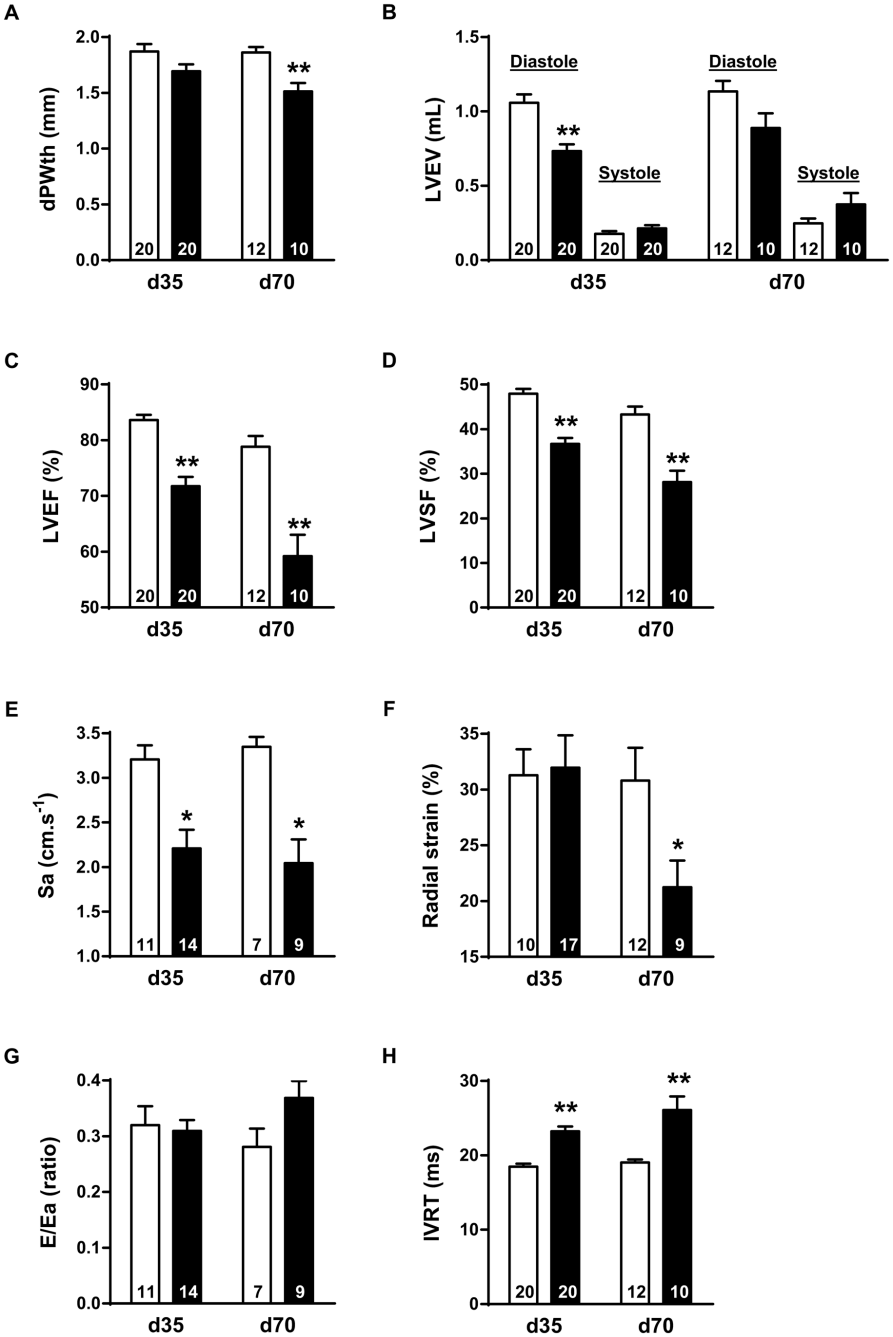
*In vivo* cardiac parameters obtained by echocardiography-Doppler in Ctrl and Dox-CM rats. Ctrl are showed in empty bar and Dox-CM in full bar. Values are means ± sem *: *P*<0.05 *vs* respective Ctrl **: *P*<0.001 *vs* respective Ctrl. d35: day 35, d70: day 70, Dox-CM: Doxorubicin-induced cardiomyopathy, dPWth: diastolic posterior wall thickness; E: E wave velocity; Ea: peak velocity of basal and lateral walls in early diastole; IVRT: isovolumic relaxation time; LV: left ventricle; LVEF: left ventricular ejection fraction; LVEV: left ventricular end volume; LVSF: left ventricular shortening fraction; Sa: peak velocity of basal and lateral walls in systole.

The dPWth decreased in Dox-CM compared to Ctrl hearts at d35 (−9.5±3.3%, *P* = 0.055) and was significantly thinner at d70 (−20.5±4.4%, *P*<0.001) (Figure 3A). LVESV was similar in both groups at d35 as at d70 (*P* = 0.199 and *P* = 0.116, respectively). On the contrary, LVEDV was reduced in Dox-CM compared to Ctrl at d35 (−12.6±1.9%, *P*<0.001) as at d70 (−21.7±8.7%, *P* = 0.050) (Figure 3B).

Compared to Ctrl, Dox-CM LVs were characterized at d35 by mild but significant decreases in LVEF (-14.2±2.0%; *P*<0.001) and LVSF (−23.4±2.7%; *P*<0.001), that were more pronounced at d70 (−25.2±6.2%, *P*<0.001 and −34.9±7.4%, *P*<0.001, respectively) (Figures 3C, D). Moreover, Dox-CM hearts presented an alteration in longitudinal deformation, since Sa was decreased at d35 (−31.2±6.5%; *P* = 0.001) as at d70 (−48.6±10.3%, *P*<0.001) (Figure 3E) whereas radial deformation was only observed at d70 (−38.7±4.9%, *P* = 0.031) (Figure 3F). In Dox-CM rats, IVRT value was significantly increased at d35 (+25.7±3.4%, *P*<0.001) as at d70 (+31.1±10.6%, *P* = 0.003) (Figure 3G) albeit the LV filling pressures, evaluated by E/Ea ratio, were similar in Ctrl and Dox-CM rats at both d35 and d70 (*P* = 0.776 and *P* = 0.071, respectively) (Figure 3H).

### Cardiac Function Obtained by Left Ventricle Catheterization

As our study consists to identify molecular target at early stage of Dox-CM, we only performed LV catheterization at d35. Basal heart rate, measured before all pressure acquisitions, was increased in Dox-CM rats compared to Ctrl (*P* = 0.007) (Table 3). Dox-CM rats presented a decreased Tau (−10.0±2.2%; *P* = 0.009) albeit dP/dt_max_ and dP/dt_min_ were unchanged (Table 3). LVEDP was unchanged between both groups (*P* = 0.216).

**Table 3:** *In vivo* cardiac parameters obtained by LV catheterization in Ctrl and Dox-CM rats, at d35.

Parameters		Ctrl (n = 10)	Dox-CM (n = 16)
**Heart rate** (bpm)		325±10	366±9*
**LV contractility**	**dP/dt_max_** (mmHg.s^−1^)	5782±425	5749±330
**LV diastolic function**	**Tau** (ms)	9.30±0.26	8.38±0.20*
	**dP/dt_min_** (mmHg.s^−1^)	−5649±511	−5861±306
**LV loading conditions**	**LVEDP** (mmHg)	8.10±1.03	6.69±0.60

Ctrl: control; Dox-CM: Doxorubicin-induced cardiomyopathy; dP/dt_max_: left ventricular contraction velocity; dP/dt_min_: left ventricular relaxation velocity; LV: left ventricle; LVEDP: left ventricular end diastolic pressure; Tau: index of left ventricular relaxation constant. Values are means ± sem. *: *P*<0.05 *vs* Ctrl.

### β_3_-adrenoceptor Expression and Function

**Figure 4:**
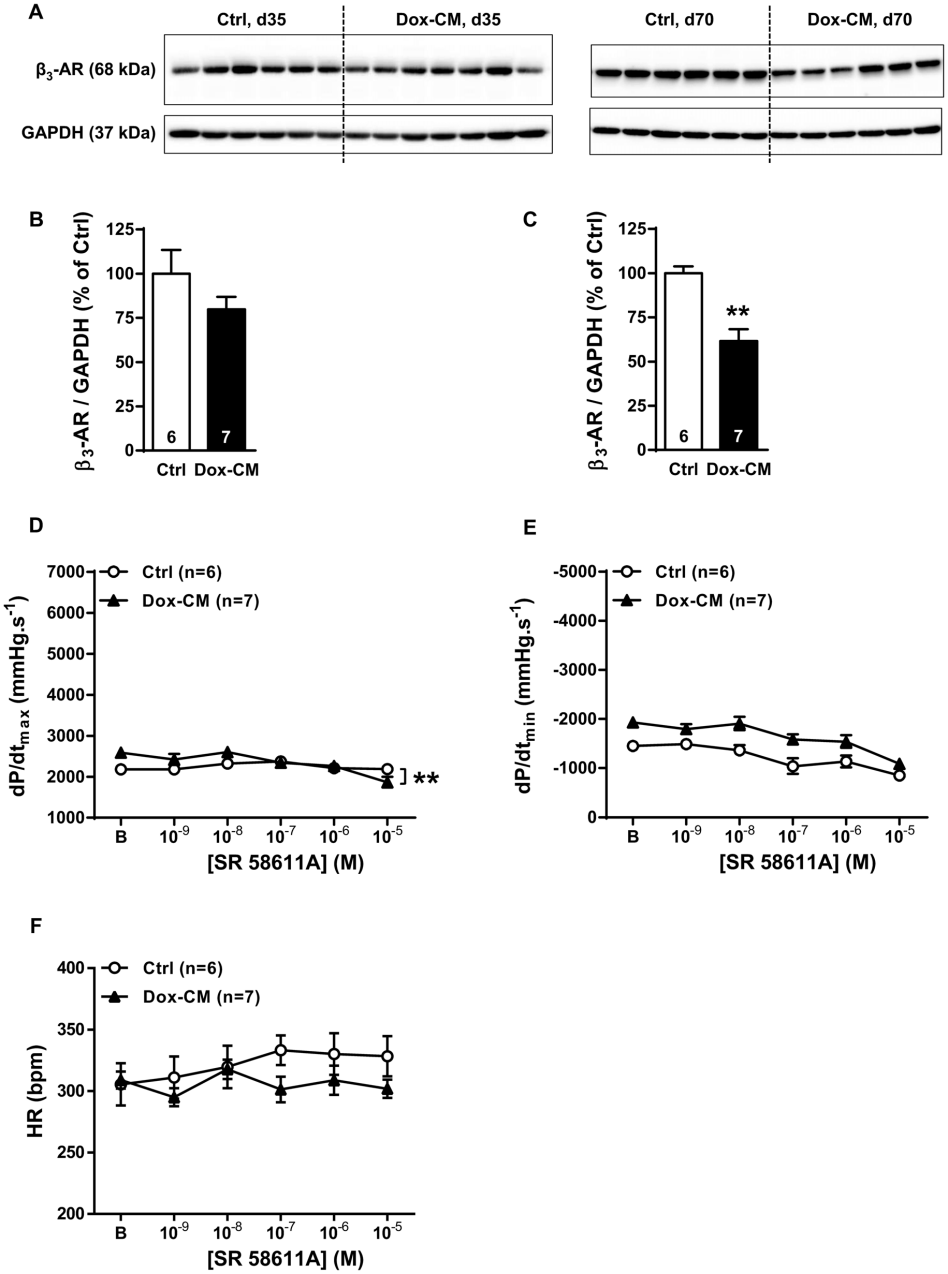
β_3_-AR expression and function in Dox-CM hearts. A. Representative β_3_-AR immunoblotting at days 35 (d35) and 70 (d70). B. β_3_-AR protein quantification at day 35. C. β_3_-AR protein quantification at day 70. Protein levels were quantified using Amersham ImageQuant RT-ECL camera (GE Healthcare). The band signals were assessed by densitometry with ImageQuant TL software (GE Healthcare) and a ratio to the corresponding GAPDH band intensity was calculated. D. Cardiac inotropic (dP/dt_max_), E. lusitropic (dP/dt_min_) and F. chronotropic (HR) effects of increasing concentrations of SR 58611A (10^−9^ to 10^−5^ M) were evaluated on isolated perfused hearts. **: *P*<0.001 *vs* Ctrl. Ctrl: control, Dox-CM: Doxorubicin-induced cardiomyopathy.

Using western-blot, we detected β_3_-AR protein at a band of 68 kDa (Figure 4A). At d35, the detected band presented a similar intensity in Dox-CM and Ctrl (*P* = 0.192), whereas, at d70, β_3_-ARs were down-expressed in Dox-CM hearts compared to Ctrl hearts (−37.2±7.9%; *P*<0.001) (Figures 4A–C). In Ctrl isolated heart, at d35, β_3_-AR stimulation performed by cumulative increasing concentrations of SR 58611A produced no significant effect on heart rate and dP/dt_max_ at any concentration (*P* = 0.239 and *P* = 0.385, respectively) (Figures 4D, F) and decreased dP/dt_min_ only at the higher concentration of SR 58611A (10^−5^M) (*P* = 0.004) (Figure 4E). Compared to Ctrl hearts, β_3_-AR stimulation in Dox-CM hearts produced a significant decrease in dP/dt_max_ only at the higher concentration of SR 58611A (10^−5^M) (*P*<0.001) (Figure 4D) and had no effect on dP/dt_min_ nor heart rate (*P* = 0.406 and *P* = 0.107, respectively) (Figures 4E, F).

### β_1_-adrenoceptor Expression and Function

**Figure 5:**
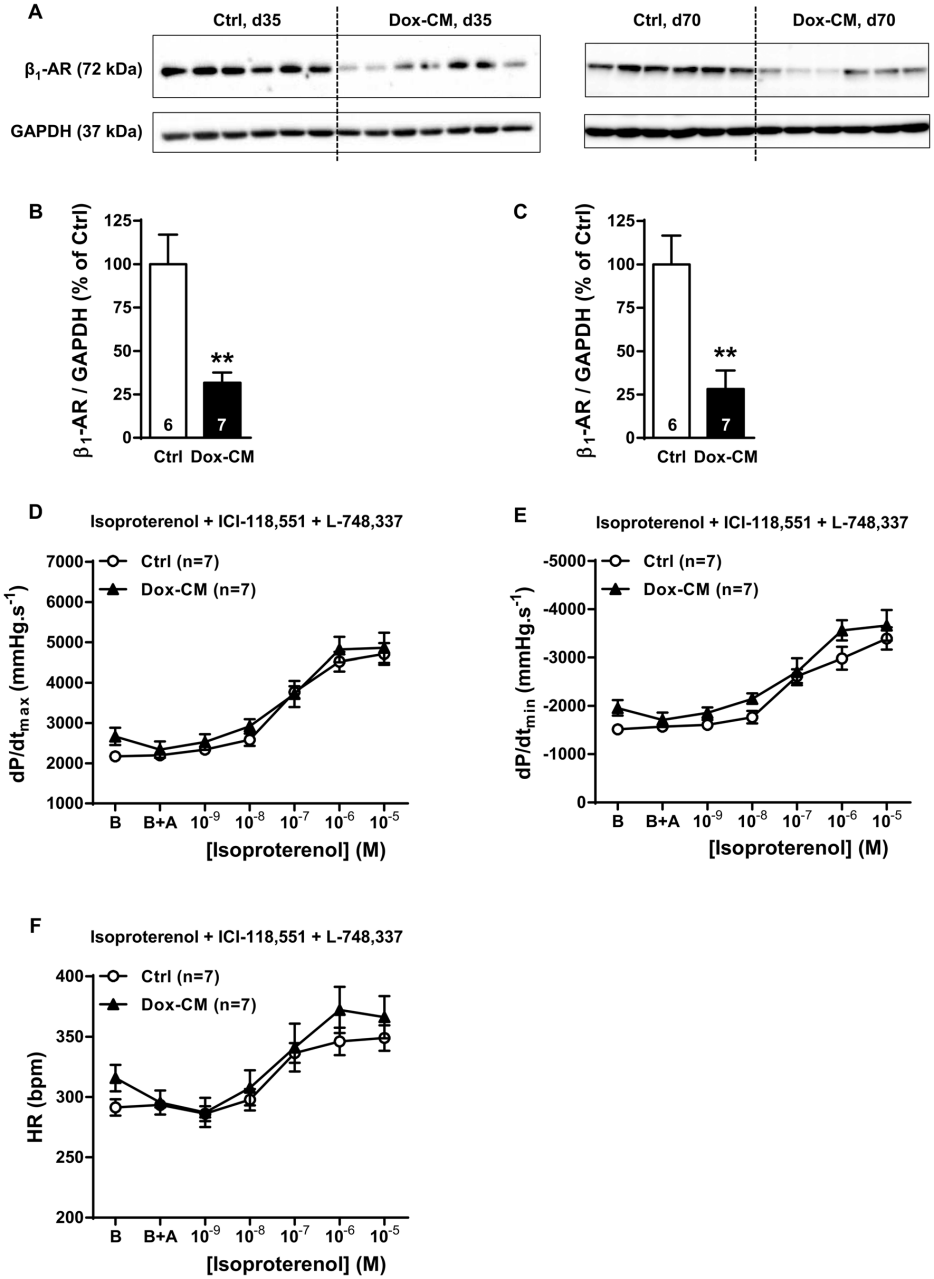
Impaired β_1_-AR expression in Dox-CM hearts was not associated to an impaired cardiac β_1_-AR function. A. Representative β_1_-AR immunoblotting at days 35 (d35) and 70 (d70). B. β_1_-AR protein quantification at day 35. C. β_1_-AR protein quantification at day 70. Protein levels were quantified using Amersham ImageQuant RT-ECL camera (GE Healthcare). The band signals were assessed by densitometry with ImageQuant TL software (GE Healthcare) and a ratio to the corresponding GAPDH band intensity was calculated. D. Cardiac inotropic (dP/dt_max_), E. lusitropic (dP/dt_min_) and F. chronotropic (HR) effects of increasing concentrations of isoproterenol (10^−9^ to 10^−5^ M) in the presence of 10^−6^ M ICI-118,551 a β_2_-AR antagonist and 10^−6^ M of L-748,337 a β_3_-AR antagonist were evaluated on isolated perfused hearts. B: Baseline; B+A; Baseline in presence of antagonists. **: *P*<0.001 *vs* Ctrl. Ctrl: control, Dox-CM: Doxorubicin-induced cardiomyopathy.

By western-blot, the antibody directed against β_1_-AR detected a band at 72 kDa (Figure 5A) whose intensity was significantly decreased in Dox-CM LV at d35 by −68.2±6.1% (*P* = 0.002 *vs* Ctrl) (Figure 5B) and at d70 by −75.3±14.0% (*P*<0.001 *vs* Ctrl) (Figure 5C). In Ctrl heart, at d35, β_1_-AR stimulation induced a concentration-dependant increase in dP/dt_max_, dP/dt_min_ and heart rate with a maximal effect observed at a concentration of 10^−5^ M (*P*<0.001, each) (Figures 5D–F). The heart rate and the positive inotropic and lusitropic effects were unchanged in Dox-CM isolated hearts compared to Ctrl hearts (*P* = 0.544, *P* = 0.772 and *P* = 0.667, respectively) (Figures 5D–F).

### β_2_-adrenoceptor Expression and Function

**Figure 6:**
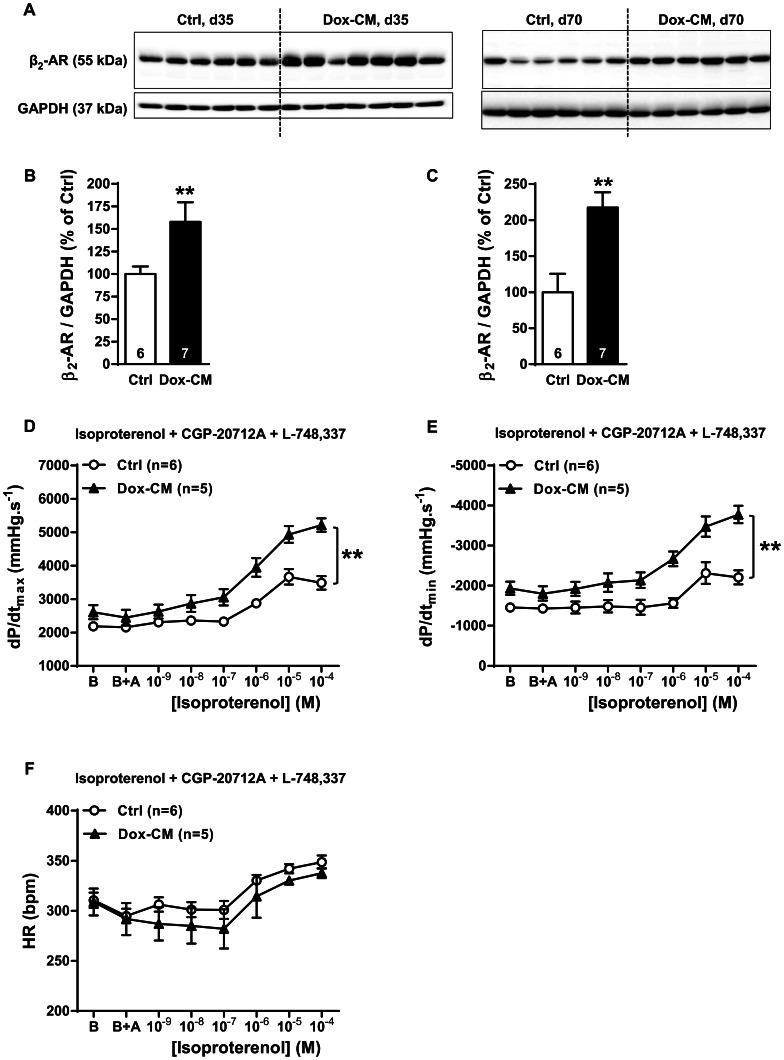
β_2_-AR expression and function are increased in Dox-CM hearts. A. Representative β_2_-AR immunoblotting at days 35 (d35) and 70 (d70). B. β_2_-AR protein quantification at day 35. C. β_2_-AR protein quantification at day 70. Protein levels were quantified using Amersham ImageQuant RT-ECL camera (GE Healthcare). The band signals were assessed by densitometry with ImageQuant TL software (GE Healthcare) and a ratio to the corresponding GAPDH band intensity was calculated. D. Cardiac inotropic (dP/dt_max_), E. lusitropic (dP/dt_min_) and F. chronotropic (HR) effects of increasing concentrations of isoproterenol (10^−9^ to 10^−4^ M) in presence of 10^−6^ M of CGP-20712A a β_1_-AR antagonist and 10^−6^ M of L-748,337 a β_3_-AR antagonist were evaluated on isolated perfused hearts. B: Baseline; B+A; Baseline the in presence of antagonists. **: *P*<0.001 *vs* Ctrl; Ctrl: control, Dox-CM: Doxorubicin-induced cardiomyopathy.

By western-blot, the antibody directed against β_2_-AR detected a band at 55 kDa (Figure 6A) whose intensity was significantly increased in Dox-CM LV at d35 by +57.9±21.6% (*P* = 0.039 *vs* Ctrl) (Figure 6B) and at d70 by +173.7±35.1% (*P*<0.001 *vs* Ctrl) (Figure 6C). At d35, β_2_-AR stimulation induced a similar concentration-dependant increase of heart rate between Ctrl and Dox-CM hearts (*P* = 0.695) (Figure 6F). In Ctrl isolated hearts, β_2_-AR stimulation induced a concentration-dependant increase of dP/dt_max_ and dP/dt_min_ (*P*<0.001 and *P* = 0.005, respectively) (Figures 6D, E) but at a lesser extent than β_1_-AR one. In Dox-CM hearts, the maximal effects induced by β_2_-AR stimulation on dP/dt_max_ and dP/dt_min_, compared to Ctrl, were both increased by +108.0±18.2% (*P*<0.001) and +155.3±26.8% (*P*<0.001), respectively (Figures 6D, E), and reached a similar level than this produced by β_1_-AR stimulation.

### Gs Protein Expression and Adenylyl Cyclase Stimulation

**Figure 7:**
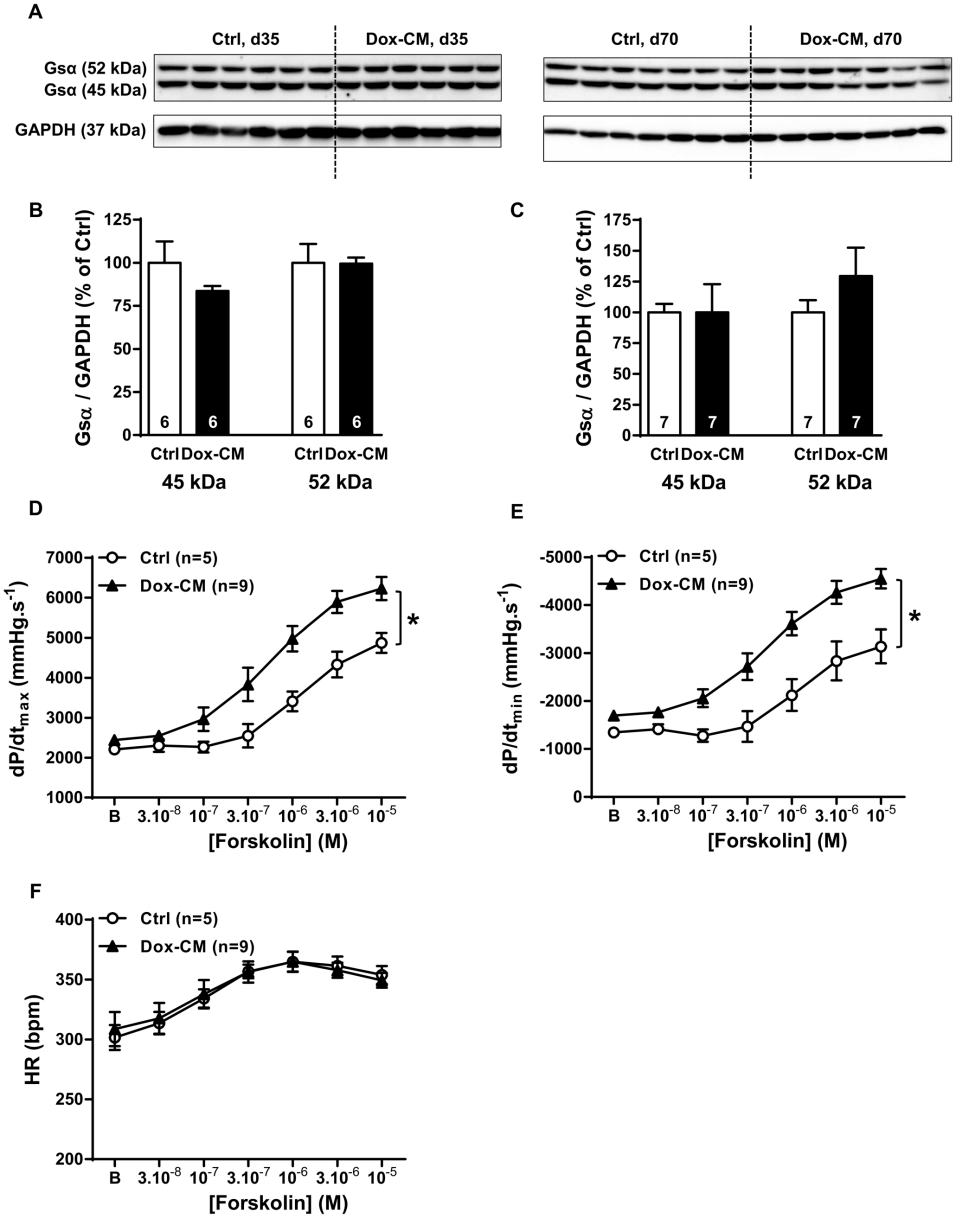
Gsα protein expression and forskolin response. **A.** Representative Gsα immunoblotting at days 35 (d35) and 70 (d70). **B.** Gsα protein quantification at day 35. **C.** Gsα protein quantification at day 70. Protein levels were quantified using Amersham ImageQuant RT-ECL camera (GE Healthcare). The band signals were assessed by densitometry with ImageQuant TL software (GE Healthcare) and a ratio to the corresponding GAPDH band intensity was calculated. **D.** Cardiac inotropic (dP/dt_max_), **E.** lusitropic (dP/dt_min_) and **F.** chronotropic (HR) effects of increasing concentrations of the adenylyl cyclase activator forskolin (3.10^−8^ to 10^−5^ M) were evaluated on isolated perfused hearts. *: *P*<0.05 *vs* Ctrl. Ctrl: control, Dox-CM: Doxorubicin-induced cardiomyopathy.

By western-blot, we detected two bands for Gsα protein expression: a short one at 45 kDa and a long one at 52 kDa (Figure 7A). The expression of both forms was unchanged in Dox-CM hearts at both d35 and d70 (Figures 7B, C). In Ctrl and Dox-CM isolated hearts, at d35, forskolin induced a similar concentration-dependant increase of heart rate between Ctrl and Dox-CM hearts (*P* = 0.941) (Figure 7F). In Ctrl group, forskolin induced a concentration-dependant increase of dP/dt_max_ (*P*<0.001) and dP/dt_min_ (*P* = 0.010) (Figures 7D, E). In Dox-CM isolated hearts compared to Ctrls, the forskolin effects on dP/dt_max_ and dP/dt_min_ were increased by +43.2±10.3% (*P* = 0.004) and +61.1±10.7% (*P* = 0.002), respectively (Figures 7D, E).

### Gi Protein Expression and its Involvement in β_2_-AR Cardiac Contractility

**Figure 8:**
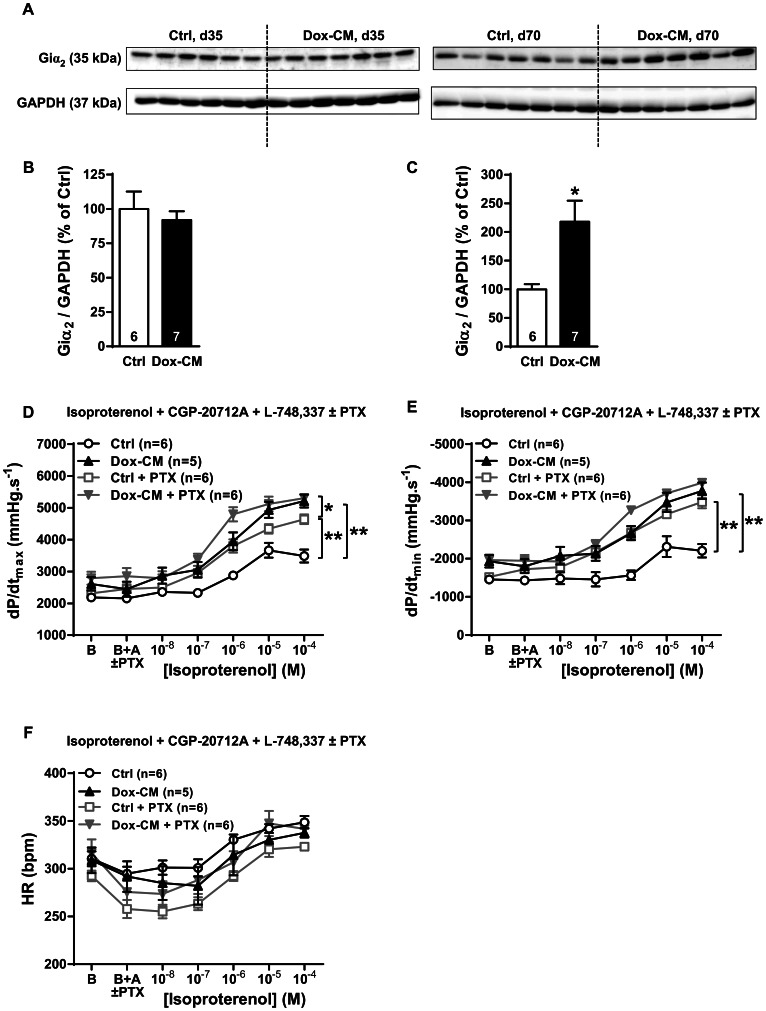
Gi protein expression and involvement in β_2_-AR cardiac contractility. **A.** Representative Giα_2_ immunoblotting at days 35 (d35) and 70 (d70). **B.** Giα_2_ protein quantification at day 35. **C.** Giα_2_ protein quantification at day 70. Protein levels were quantified using Amersham ImageQuant RT-ECL camera (GE Healthcare). The band signals were assessed by densitometry with ImageQuant TL software (GE Healthcare) and a ratio to the corresponding GAPDH band intensity was calculated. **D.** Cardiac inotropic (dP/dt_max_), **E.** lusitropic (dP/dt_min_) and **F.** chronotropic (HR) effects of increasing concentrations of isoproterenol (10^−9^ to 10^−4^ M) in the presence of 10^−6^ M of CGP-20712A and 10^−6^ M of L-748,337 were evaluated on isolated perfused hearts pre-treated or not with 4 µg.L^−1^ of pertussis toxin (PTX) a Gi protein inhibitor. B: Baseline; B+A; Baseline in the presence of antagonists. *: *P*<0.05 *vs* Ctrl; **: *P*<0.001 *vs* Ctrl. Ctrl: control, Dox-CM: Doxorubicin-induced cardiomyopathy.

Giα_2_ protein expression was detected by one band of 35 kDa (Figure 8A) whose intensity was similar both in Ctrl and Dox-CM hearts at d35 (*P* = 0.184 *vs* Ctrl) (Figure 8B), but was increased in Dox-CM hearts at d70 by +118.1±37.6% (*P* = 0.009 *vs* Ctrl) (Figure 8C). *Ex vivo*, at d35, Gi protein inhibition by PTX pre-treatment produced no effect in heart rate in both groups (*P* = 0.346) (Figure 8F). PTX-pretreatment induced a significant increase of β_2_-AR stimulation in Ctrl isolated hearts (dP/dt_max_: +64.4±9.8%; *P*<0.001; dP/dt_min_: +128.0±20.8%; *P*<0.001) (Figures 8D, E) but induced no change of these two parameters in Dox-CM hearts (*P* = 0.173 and *P* = 0.451, respectively) (Figures 8D, E).

## Discussion

This study demonstrated for the first time in Dox-cardiotoxicity, β_2_-AR expression was increased at the early stage of the pathology, with an increase of β_2_-AR-induced contractility. Furthermore, β_1_-AR function was preserved in spite of decreased β_1_-AR protein expression and could be explained by an increase of AC expression and/or activity as illustrated by an increased forskolin-induced contractility in isolated hearts from Dox-CM rats.

In our study, the body weight of Dox-CM rats was smaller than in Ctrl one as previously described by other studies [8], [24]. This has been related to a decrease in food consumption [25], [26]. In our experimental conditions, we have, 35 days after the beginning of Dox-treatment, 25% of mortality and rats presented a large amount of ascites. Those observations were in agreement with other studies that reported a mortality rate fluctuating between 19% and 45% [27], [28] and a volume of ascites generally varying from 30 mL to 140 mL [24], [28]–[30]. However, as Dox induced a multi-organ toxicity, it could be not excluded that mortality and ascites observed in rats could be due, at least in part, to liver and kidney damages. Seventy days after the beginning of Dox-treatment, mortality rate increased to 58% and ascites was still observed. Very few studies reported long term effects of Dox-treatment and data on mortality and ascites are very heterogeneous. Whereas a study observed 50% mortality rate 6 weeks after the last Dox-injection, associated with a very large amount of ascites [25] others did not observed mortality eight weeks after the last Dox-injection [11]. Also, others did not reported mortality and observed a decrease in ascites amount between days 40 and 70 [26].

During echocardiographic acquisitions, heart rate was monitored in order to have similar heart rate in both groups, allowing a comparison of echocardiographic parameters. At d35, Dox-CM rats presented a slight alteration of systolic function as illustrated by a mild decrease in LVEF and LVSF and in LV longitudinal deformation. Dox-CM rats presented also a mild diastolic dysfunction as suggested by the increased IVRT and Tau values. At d70, decreases in LVEF, LVSF and LV longitudinal deformation were exacerbated whereas diastolic dysfunction was unchanged. As diastolic dysfunction frequently occurs before systolic dysfunction [31]–[33], we could suggest that in our animal model, at d35, systolic function just began to be altered, meaning that this model corresponds to an early chronic Dox-cardiotoxicity. It is important to note that our results are very close to that observed in women who received anthracycline-treatment for breast cancer. Indeed, these patients had preserved LVEF compared to controls but a significantly reduced longitudinal strain with preserved radial contractility [34].

In human, Dox administered to adults (for breast cancer, for example) is well known to induce chronic cardiotoxicity where cardiac morphologic and functional alterations are close to those of dilated cardiomyopathy [3]. However, when administered in children, Dox can induce restrictive cardiomyopathy with normal LV dimensions [35]. Those findings, are consistent with those of other long-term follow-up studies conducted in other groups of anthracycline-treated childhood cancer survivors [36]. In our study, Dox was administered to young rats (7 week-old). At d35, we observed diastolic dysfunction with normal systolic function that could support the hypothesis of a restrictive-cardiomyopathy. At d70, the development of systolic dysfunction could suggest restrictive cardiomyopathy worsening with increase LV dimensions. Indeed, although LVEDDs were not statistically different between Ctrl and Dox-CM rats at d70, it is important to note that LV dPWth was significantly decreased in Dox-CM compared to Ctrl rats, strengthening the notion of LV morphologic changes in those rats. We could hypothesize that a period of 56 days post-treatment was a too short duration of the disease evolution to observe a significant LV dilation in Dox-CM rats. Indeed, in human HF, the dilation is considered as the last stage of ischemic or non ischemic cardiomyopathy evolution as in Dox-CM. In this latter disease, it is well known that cardiomyopathy and thus LV dilation occur up to decades after exposure [37].

In our Dox-CM model, we reported a remodeling in β-AR expression and function. Three weeks after last Dox-injection (d35), β_3_-AR protein expression was unchanged and a slight negative inotropic effect was obtained in Dox-CM hearts at the higher concentration of SR 58611A (10^−5^ M). Our data suggest that in early chronic Dox-cardiotoxicity, β_3_-AR could not play a major role in the cardiac alterations. At a later stage (d70), we observed a decreased β_3_-AR protein expression in Dox-CM hearts albeit another team reported an overexpression of β_3_-AR protein at the same stage [11]. Several hypotheses could explain those discrepancies. First, the rat strain used in the study is different (Wistar *vs* Sprague-Dawley) and several studies reported that protein expression could be different between Sprague-Dawley and Wistar rats [38], [39] albeit no study compared β-AR subtypes expression between these two rat strains. Second, in the Sun’s study, cardiac β-ARs expressions were assessed in Ctrl rats compared to Dox-treated rats which were also Sham-castrated (scrotum incision); no surgery being performed in Ctrl rats. Therefore, β-AR protein expressions were assessed in a Dox-CM model different from our one used in our study.

In our Dox-CM model, β_1_-AR protein expression was decreased at both stages. This result is in agreement with those reported for β_1_-AR protein expression both in rabbits [40] and in rats [8], [11]. Surprisingly, albeit a decreased β_1_-AR expression at d35, the cardiac β_1_-AR responses were preserved. Several hypotheses could explain this feature. Firstly, a β_1_-AR reserve could be present and recruited in such conditions and, secondly, an increase in β_1_-AR signaling pathway can be evoked. We showed that Dox-treatment did not change Gsα protein expression, as it was reported in other study [8], [11], but increased forskolin response, suggesting an increase in AC expression and/or activity. This surprising data contrasts with other studies which reported a decreased AC activity [41], [42] or no change [43]. However, those differences seem to be due to the cardiotoxicity level since the Dox cumulative doses used were 24, 24.75 and 6 mg.kg^−1^, respectively. Thus, the increased AC response to forskolin observed in our study could be a compensatory mechanism at early stage of Dox-cardiotoxicity to maintain cardiac contractility and could explain, at least in part, the preserved β_1_-AR response. However, as β_1_-ARs are involved in pro-apoptotic effects, β_1_-AR could be also responsible for some Dox cardiotoxic effects as previously suggested [9], [10].

Finally, we reported, for the first time, in chronic Dox-cardiotoxicity, a β_2_-AR protein overexpression. At d35, in Ctrl isolated heart, specific β_2_-AR stimulation induced positive inotropic and lusitropic responses but to a lesser extent than β_1_-AR one. This lower response could be explained by a more compartmentalized cAMP signaling of β_2_-AR [44]. Surprisingly, in Dox-CM hearts, β_2_-AR inotropic effect was increased and reached a similar level than β_1_-AR stimulation. This increase could be due to several mechanisms. Firstly, we show that β_2_-AR protein expression was increased in Dox-CM LV and could lead to an increased β_2_-AR response. Secondly, cardiac β_2_-AR could be linked both to Gs and Gi proteins [45], [46]. In Dox-CM LV, Giα_2_ protein expression was not modified at d35 but it was overexpressed at d70 of Dox-CM, as reported previously at this latter stage by Sun *et al*. [11]. Surprisingly, at d35, in Ctrl hearts, the Gi inhibition by PTX allowed to increase β_2_-AR response in order to reach the same level that obtained in Dox-CM in the absence of PTX treatment, whereas Gi inhibition in Dox-CM hearts slightly increased β_2_-AR response. These data suggest that, in Dox-CM, β_2_-AR was mainly linked to Gs.

To know whether enhanced β_2_-AR/Gs signaling observed in our study is a beneficial compensatory mechanism or a detrimental effect is still to be determined. To support the hypothesis of a beneficial effect of β_2_-AR agonist, it has been shown that β_2_-AR/Gi signaling can mediate cardiac protective effects by activating cell surviving pathway [10], [47], [48], and also *in vivo* studies reported beneficial effects of β_2_-agonist therapy in HF [49]–[54]. Concerning a putative detrimental effect of β_2_-AR, it could be proposed to use β_2_-AR blocker. However, it is important to note that β_2_-AR antagonists by blocking other physiological functions regulated by β_2_-AR, could also have detrimental effects: (i) a vasoconstriction because β_2_-AR is one of the most important vascular β-AR subtype whose activation induces a vasodilation in several vascular beds (including coronary vessels), (ii) a bronchoconstriction due to the reduction of bronchodilation induced by pulmonary β_2_-AR,…

Thus, we rather suggest that further experiments must be performed to identify the most suitable effector of the cardiac β_2_-AR pathway. Then, according to the results of this next important study, we hope that we could propose a new therapeutic target for Dox-CM.

In conclusion, we have shown for the first time in rat Dox-CM, an increase of β_2_-AR protein expression at early stage of the pathology, associated to an increased contractility. Furthermore, we observed a preserved β_1_-AR function albeit β_1_-AR was reduced. Clinical studies have suggested no beneficial effect of metoprolol, a β_1_-AR blocker, in Dox-treated lymphoma patients [15] but a beneficial effect of nebivolol, a β_1_-AR blocker associated to a β_3_-AR agonistic properties against anthracycline-induced cardiomyopathy in patients with breast cancer [55]. However, the clinical roles of β_2_-AR remain to elucidate in order to determine whether β_2_-AR activation or blockade could prevent cardiac alterations due to anthracycline.
